# Identification and validation of pyroptosis-related genes in Alzheimer’s disease based on multi-transcriptome and machine learning

**DOI:** 10.3389/fnagi.2025.1568337

**Published:** 2025-05-14

**Authors:** Yuntai Wang, Yilin Li, Lu Zhou, Yihuan Yuan, Chuanfei Liu, Zimeng Zeng, Yuanqi Chen, Qi He, Zhuoze Wu

**Affiliations:** ^1^Institute of Basic Medicine, North Sichuan Medical College, Nanchong, China; ^2^School of Clinical Medicine, North Sichuan Medical College, Nanchong, China; ^3^School of Integrated Traditional Chinese and Western Clinical Medicine, North Sichuan Medical College, Nanchong, China; ^4^School of Medical Imaging, North Sichuan Medical College, Nanchong, China; ^5^School of Nursing, North Sichuan Medical College, Nanchong, China

**Keywords:** Alzheimer’s disease, pyroptosis, machine learning, bioinformatics, immune infiltration, regulatory network

## Abstract

**Background:**

Alzheimer’s disease (AD) progression is characterized by persistent neuroinflammation, where pyroptosis—an inflammatory programmed cell death mechanism—has emerged as a key pathological contributor. However, the molecular mechanisms through which pyroptosis-related genes (PRGs) drive AD pathogenesis remain incompletely elucidated.

**Methods:**

We integrated multiple transcriptomes of AD patients from the GEO database and analyzed the expression of PRGs in combined datasets. Machine learning algorithms and comprehensive bioinformatics analysis (including immune infiltration and receiver operating characteristic (ROC)) were applied to identify the hub genes. Additionally, we validated the expression patterns of these key genes using the expression data from AD mice and constructed potential regulatory networks through time series and correlation analysis.

**Results:**

We identified 91 PRGs in AD using the weighted gene co-expression network analysis (WGCNA) and differentially expressed genes analysis. By application of the protein–protein interaction and machine learning algorithms, seven pyroptosis feature genes (CHMP2A, EGFR, FOXP3, HSP90B1, MDH1, METTL3, and PKN2) were identified. Crucially, MDH1 and PKN2 demonstrated superior performance in terms of immune cell infiltration, ROC curves, and experimental validation. Furthermore, we constructed the long non-coding RNA and mRNA (lncRNA-mRNA) regulatory network of these characteristic genes using the gene expression profiles from AD mice at varying ages, revealing the potential regulatory mechanism in AD.

**Conclusion:**

This study provides the first comprehensive characterization of pyroptosis-related molecular signatures in AD. Seven hub genes were identified, with particular emphasis on MDH1 and PKN2. Their superior performances were validated through comprehensive bioinformatic analysis in both patient and mouse transcriptomes, as well as the experimental data. Our findings establish foundational insights into pyroptosis mechanisms in AD that may inform novel treatment strategies targeting neuroinflammatory pathways.

## 1 Introduction

Alzheimer’s disease (AD) represents the most prevalent neurodegenerative disorder, clinically manifested by progressive deterioration of memory functions (Hodson, 2018). Disease progression is accompanied by a constellation of neurological deficits, including language impairment, affective disturbances, spatial disorientation, and behavioral abnormalities (Gonzales et al., 2022). The age of onset of AD is predominantly above 65 years, and with the increasing trend of global population aging, its prevalence is expected to rise (Raggi et al., 2022). Current therapeutic limitations underscore the urgent need for novel treatment targets, as no therapies exist to halt or reverse AD pathogenesis.

Neuronal dysfunction and loss constitute fundamental contributors to AD-associated cognitive decline. Two pathological hallmarks, amyloid-β (Aβ) plaques and neurofibrillary tangles (NFTs), precede clinical symptom onset (Mattson, 2004). It has been demonstrated that Aβ accumulation triggers microglial activation, initiating neuroinflammatory cascades that culminate in neuronal death (Podlesny-Drabiniok et al., 2020; Leng and Edison, 2021). These evidence suggest that inflammation-mediated neuronal demise may represent a critical therapeutic target. Pyroptosis is a novel pattern of programmed cell death (PCD) characterized by inflammatory necrosis that has been confirmed in recent years (Rao et al., 2022). A multitude of studies have demonstrated that pyroptosis plays a role in the progression of neurological, metabolic, cardiovascular, and infectious diseases (Hu et al., 2022; Moonen et al., 2023; Yarovinsky et al., 2023; Wei et al., 2022; Al Mamun et al., 2024). Classical pyroptosis involves inflammasome-mediated caspase-1 activation, leading to gasdermin D (GSDMD) cleavage and pore formation in cell membranes (Shi et al., 2015). This process facilitates the release of pro-inflammatory cytokines IL-1β and IL-18, driving neuroinflammation (Rao et al., 2022).

Emerging evidence positions pyroptosis as a potential therapeutic target in AD. A recent review outlines the clues of Aβ accumulation-induced NOD-like receptor family pyrin domain-containing proteins 1 and 3 (NLRP1 and NLRP3) inflammasome-dependent pyroptosis in AD (Hu et al., 2024). Experimental reduction of NLRP1 or caspase1 expression in APP/PS1 (an AD model) mice attenuated the Aβ deposition, reduced neuronal pyroptosis, and improved cognitive deficits (Tan et al., 2014; Flores et al., 2022). NLRP3 has also been identified as a contributing factor in the development of AD pathology, with a main role in the mediation of microglia pyroptosis (Cai et al., 2021; de Brito Toscano et al., 2021). These studies indicate that pyroptosis could be a promising direction of investigation and a potential therapeutic target for AD. In addition, the non-classical signaling pathway, apoptotic caspases-mediated pathway, and granzymes-mediated pathways have been recognized in pyroptosis (Wei et al., 2022). The mechanisms mediated by these pathways are independent of the classical inflammasome complex and warrant further investigation in AD. Moreover, there is a lengthy interval between the persistence of neuroinflammation and the onset of cognitive impairment in AD (Gonzales et al., 2022). Systematic identification of differentially expressed pyroptosis-related genes (PRGs) across disease stages could unveil molecular drivers of AD progression. Multi-transcriptomic integration combined with machine learning may provide novel insights into pyroptosis-mediated pathophysiology.

**Figure 1:**
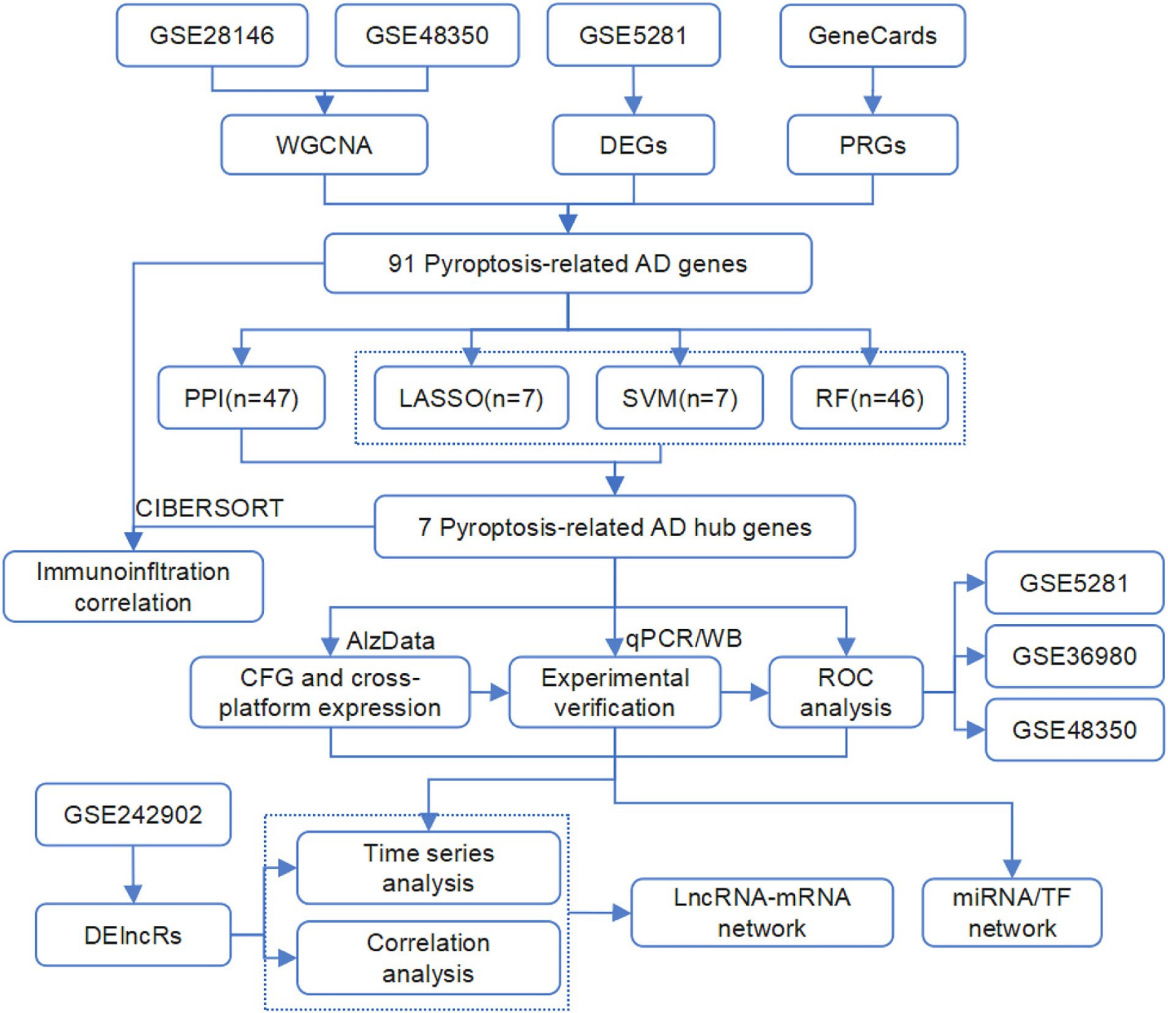
The flowchart of the analysis process in this study.

In this study, we conducted a comprehensive analysis of expression data from AD patients and normal controls across multiple GEO datasets. We employ weighted gene co-expression network analysis (WGCNA), differential expression analysis, gene function analysis, and machine learning algorithms to identify hub genes. The aberrant expression of these genes was confirmed by additional AD datasets, our AD mice dataset, and molecular experiments using AD mice. In addition, our dataset comprised the expression profiles of APP/PS1 mice at different ages, which were analyzed to identify age-dependent expression profiles of the pyroptosis-AD hub genes and their potential regulatory networks. The complete workflow of the study is illustrated in Figure 1. We believe that our findings will contribute to advancing knowledge regarding the role of pyroptosis in AD.

## 2 Materials and methods

### 2.1 Data preparation and processing

The Gene Expression Omnibus (GEO) datasets ID we acquired and analyzed in this study were GSE28146 (Blalock et al., 2011), GSE48350 (Berchtold et al., 2008), GSE5281 (Liang et al., 2007), GSE36980 (Hokama et al., 2014), and GSE242902 (Wu et al., 2024). The majority of these datasets comprise assays derived from human brain tissues, including the hippocampus and cortex. Given that the samples in these datasets originate from various brain regions, to guarantee the reliability of the data post-merging and analysis, we selected the hippocampus, which is present in all datasets, for subsequent analysis. Among them, the datasets of GSE5281, GSE28146, and GSE48350 shared the same annotation platform GPL570, and we randomly chose one of them, GSE5281, for differential expression genes analysis. The remaining two datasets (GSE28146 and GSE48350) would be combined into one dataset (containing the hippocampus of 41 AD patients and 51 control samples) for WGCNA analysis. The dataset GSE36980 was used to validate the signature genes by mapping ROC curves. Moreover, the dataset GSE242902 was our uploaded data containing a total of 18 samples of two mice groups; specifically, there are 3-, 6-, and 12-month-old APP/PS1 and wild-type (WT) mice (*n* = 3 per group). These data were used to provide multi-species validation of AD through comparative analysis across humans and mice. The hippocampi of these mice were isolated for mRNA microarray, and the protocol was as described before (Wu et al., 2024).

### 2.2 Weighted gene co-expression network analysis (WGCNA)

Weighted gene co-expression network analysis was conducted on datasets GSE28146 and GSE48350 to identify gene modules highly associated with AD. Specifically, the GSE28146 (containing the hippocampus of 22 AD patients and 8 control samples) and GSE48350 (containing the hippocampus of 19 AD patients and 43 control samples) that shared the same platform GPL570 have been combined into one matrix for WGCNA analysis after removing batch effects using the removeBatchEffect function in the Limma R package. The genes with similar patterns were grouped into a module, which was then subjected to phenotypic analysis (i.e., disease). Finally, overlapping genes between significant modules and PRGs (from the GeneCards database) were identified using Venn diagrams.

### 2.3 Differential expression gene (DEG) analysis

The data matrix of GSE5281 (containing the hippocampus of 10 AD patients and 13 control samples) was obtained from the GEO database for the DEGs analysis between AD patients and control people by the R package Limma. The data in the matrix has been subjected to a process of normalization and filtering using a quantification algorithm. DEGs between AD and control groups in hippocampus tissues were selected for identification by fold change and *p*-value, which were calculated by *t*-test, with the thresholds set at |fold change| > 2 and *p*-values <0.05. Volcanograms were plotted by https://www.bioinformatics.com.cn (last accessed on 10 October 2024), an online platform for data analysis and visualization (Tang et al., 2023). The differentially expressed mRNAs (DEmRs) or lncRNAs (DElncRs) between AD and control mice from the GSE242902 were obtained by the same calculation.

### 2.4 Identification and validation of the pyroptosis-related AD genes

The PRGs list was retrieved from the GeneCards database using the search term “pyroptosis.” Subsequently, intersection analysis was performed to identify the pyroptosis-related AD genes between (1) PRG candidates and DEGs obtained from the GSE5281 dataset, and (2) PRG candidates and module identified through WGCNA of merged datasets (GSE28146 and GSE48350). The resulting pyroptosis-related AD candidate genes were subjected to hub gene identification through integrated analysis incorporating machine learning algorithms and protein–protein interaction (PPI) analysis. We next plotted receiver operating characteristic (ROC) curves to assess the significant differences of these hub genes associated with pyroptosis between the AD and control in combined dataset and GSE36980 by using the R package ‘pROC’.

### 2.5 Protein–protein interaction (PPI) analysis

The PPI analysis was systematically constructed through the STRING database (version 10)^1^ with default parameters. Subsequent interactive visualization of the PPI network was performed using the Chiplot platform.^2^ Stringent filtering criteria were applied to identify hub interactors: nodes with degree centrality exceeding 2 and combined interaction scores >0.9 were selected as key AD-associated genes.

### 2.6 Machine learning-driven hub gene identification

Three machine learning models, namely least absolute shrinkage and selection operator (LASSO), random forest (RF), and support vector machine (SVM), were employed to analyze the hub of these pyroptosis-related AD candidate genes using the R package “Glmnet” and “Caret.” The expression of the pyroptosis-related AD gene was de-batched and merged from the datasets GSE5281, GSE28146, and GSE48350. Consensus hub genes were derived through intersecting gene lists generated by the three algorithms and visualized via Venn diagrams.

### 2.7 Gene set enrichment analysis (GSEA)

Gene set enrichment analysis (GSEA) was conducted to evaluate the trend in the distribution of genes from a pre-defined gene set in a table of genes sorted by phenotypic relevance to determine their contribution to the phenotype. In our study, we used the Gene Ontology (GO) terms as the pre-defined gene sets. Batch-corrected composite datasets derived from GSE5281, GSE28146, and GSE48350 were utilized for GSEA. Statistical significance was assessed by comparing the enrichment scores with the enrichment results generated by randomization of the gene set to derive a nominal *p*-value. The level of significance was determined by a normalized enrichment score (NES) > 0, *p* < 0.05.

### 2.8 Analysis of immune infiltration

Given the well-documented association between pyroptosis-AD genes and immune cell dynamics, we investigated the relationship between PRGs and immune cell composition using the CIBERSORT algorithm. We uploaded the combined data of GSE5281, GSE28146, and GSE48350, which were used to analyze the immune cell infiltration. Then, the “Vioplot” software package was used to visualize the differences in immune cell infiltration between the AD and control groups. In addition, the “Corrplot” software package was used to demonstrate the Spearman correlation between immune cell and gene expression.

### 2.9 Convergent functional genomics (CFG) analysis

Convergent functional genomics (CFG) framework was applied to prioritize AD candidate genes through the AlzData integrative database (Xu et al., 2018). AlzData is a one-step database of current AD data and could serve as an in-depth integrating system to integrate data of different levels and generate a prioritized gene list for further characterization. The CFG method can verify the effectiveness and confirm the AD hub genes based on the four pieces of evidence defined by the database. At the same time, the database can provide gene expression and comparison results in different brain regions by integrating multiple datasets. The platform’s cross-dataset validation capability further strengthened the biological credibility of identified AD hub genes.

### 2.10 Quantitative polymerase chain reaction (qPCR)

Following machine learning-based identification, pyroptosis-related AD hub genes were experimentally validated using the hippocampus from AD mouse models via qPCR. Primer sequences (Supplementary Table S1) were synthesized by Sangon Biotech (Shanghai, China). Total RNA was extracted from hippocampal tissues using the RNA Isolation Kit (DP501, Tiangen, China), followed by first-strand cDNA synthesis with 1 μg high-quality RNA using the 1st Strand cDNA Synthesis Kit (11141ES60, Yeason, China). Quantitative PCR amplification was performed using the qPCR Mix (11201ES08, Yeason, China). glyceraldehyde -3-phosphate dehydrogenase (GAPDH) served as the endogenous control, with target gene expression quantified using the 2^-△△CT^ method. All reactions were conducted in triplicate to ensure technical reproducibility.

### 2.11 Western blot (WB)

Western blot (WB) was performed to ascertain gene alterations at the protein level in AD mice compared to WT controls, as previously described (Wu et al., 2025). Hippocampal tissues were homogenized in ice-cold radioimmunoprecipitation assay (RIPA) buffer (P0013C, Beyotime, China) supplemented with 1 mM phenylmethylsulfonyl fluoride (PMSF; 9 μL per 1 mg tissue). After centrifugation at 12,000 × *g* for 5 min at 4°C, supernatants were collected for protein quantification. Equal amounts of protein (30 μg/lane) were separated by 10% sodium dodecyl sulfate - polyacrylamide gel electrophoresis (SDS-PAGE) and transferred onto poly (vinylidene fluoride) (PVDF) membranes. Membranes were blocked with QuickBlock Western blocking buffer (P0252, Beyotime, China) for 15 min at room temperature, followed by overnight incubation at 4°C with primary antibodies: anti-EGFR (1:1000; A00023, Boster), anti-HSP90B1 (1:1000; PB0670, Boster), anti-MDH1 (1:1000; A04262, Boster), anti-PKN2 (1:1000; A19746, Abclonal), and anti-GAPDH (1:20,000; 60,004-1-Ig, Proteintech). The membranes were incubated with the corresponding secondary antibodies for 1 h at room temperature the next day. Protein bands were visualized using enhanced chemiluminescence (ECL) reagents (P0018, Beyotime, China) and scanned by the Tannon chemiluminescence imager and measured using the ImageJ software.

### 2.12 Construction of transcription factors and miRNA regulatory networks

The prediction of target microRNAs (miRNAs) was conducted using multiple computational platforms including TargetScan, miRDB, DIANA tools, and miRwalk (McGeary et al., 2019; Chen and Wang, 2020; Paraskevopoulou et al., 2013; Sticht et al., 2018). The specific screening criteria employed were as follows: miTG score in DIANA > 0.7 for predicted genes, binding p in miRwalk > 0.8, Target Score in miRDB > 80, and all genes in TargetScan. Candidate miRNAs required co-prediction by at least three platforms. The mRNA-miRNA interaction network was constructed using Cytoscape (version 3.10.2). Transcription factor (TF) prediction was performed through KnockTF, ENCODE, and ChIP-Atlas databases (Feng et al., 2020; Consortium EP, 2011; Zou et al., 2024), with final TF candidates identified through database intersection. Regulatory relationships were visualized via Cytoscape.

### 2.13 Time series analysis and construction of lncRNA regulatory network

Time-dependent regulatory patterns of pyroptosis-related AD genes in APP/PS1 mice were analyzed using longitudinal expression profiles of target mRNAs and differentially expressed lncRNAs (DElncRNAs). DElncRNAs with mean normalized expression <6 were excluded. All the genes used for analysis were clustered into six groups by Mfuzz, a R package used for time series analysis, according to the expression profiles from the 3-, 6-, and 12-month-old AD mice (Kumar and EF, 2007). DElncRNAs co-clustered with mRNAs were designated as potential regulators. Correlation analysis was performed based on the gene expression to carry out the correlationship between mRNA and DElncRs using the Corrplot package. The *p*-value < 0.05 and |Corr. *p*-value| > 0.6 between two transcripts should be considered as highly correlation. The final lncRNA-mRNA regulatory network was visualized through Cytoscape.

### 2.14 Animals

The APP/PS1 mice (RRID: MMRRC_034832-JAX) carry two transgenes with AD-linked mutations: a chimeric mouse/human APP with the Swedish mutation and human PSEN1 lacking exon 9 (dE9) was provided by He et al. (2021). Amyloid plaque pathology was confirmed by immunohistochemistry at 6 months of age (Minkeviciene et al., 2008). Age-matched WT littermates were used as controls. All mice were bred, reared, and housed under a 12/12 h light cycle, with lights on at 8:00 am in the Laboratory Animal Center of North Sichuan Medical College in accordance with the institutional guidelines for the Care and Use of Laboratory Animals. All animal experiments were approved by the Ethics Committee of North Sichuan Medical College.

### 2.15 Statistical analysis

The data presented in the text and figures were analyzed using GraphPad Prism software, version 9.0, and expressed as the means ± standard error of the mean (SEM). For pairwise comparisons, an unpaired two-tailed Student’s *t*-test was applied based on the normality results. The level of significance was set at *p < 0.05, **p < 0.01, and ***p < 0.001. All behavioral and molecular assessments were conducted in a blinded manner by independent investigators unaware of experimental group assignments.

## 3 Result

### 3.1 WGCNA identifies AD-associated co-expression modules

**Figure 2:**
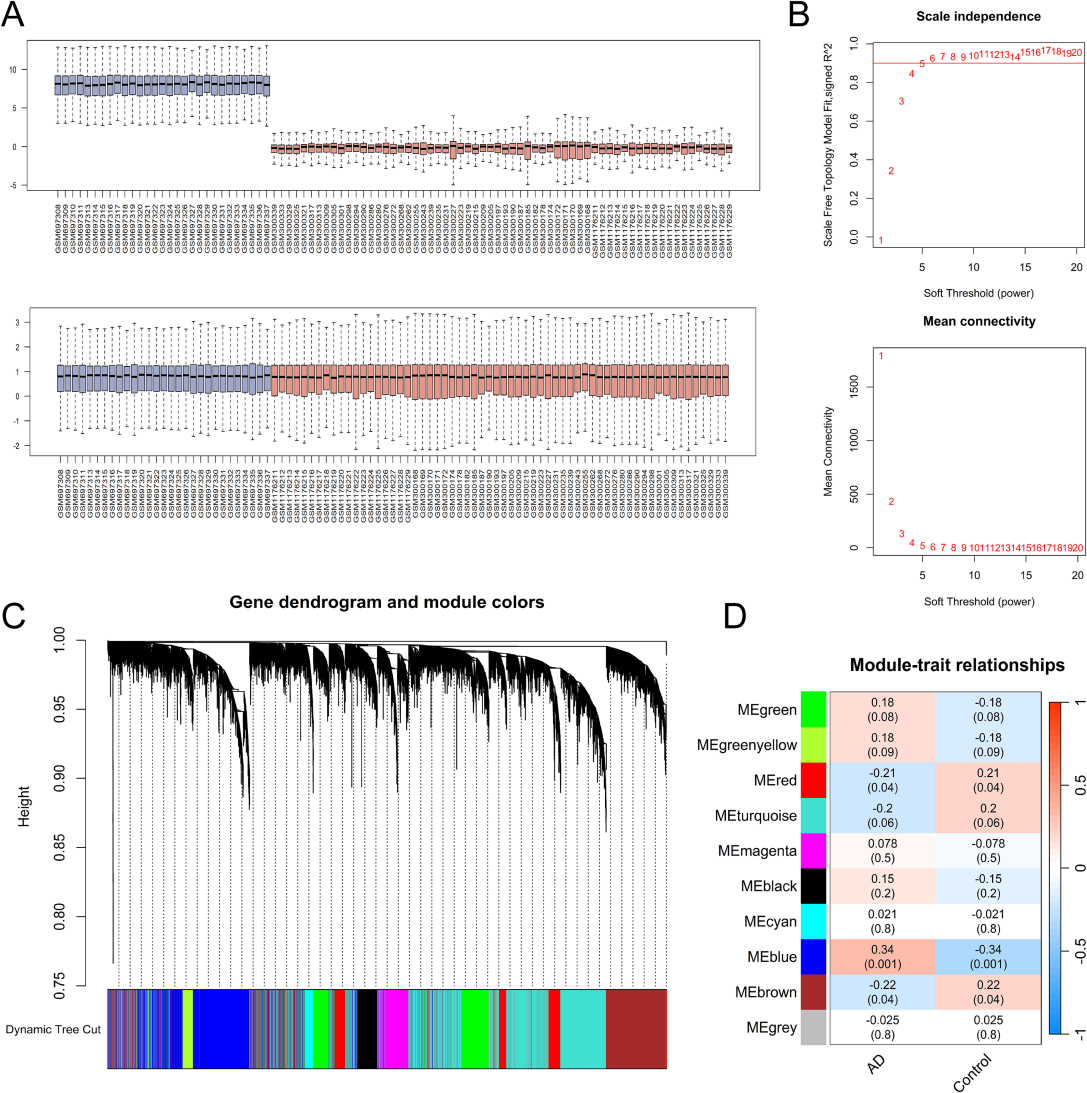
Weighted gene co-expression network analysis (WGCNA) identifies AD-associated co-expression modules. **(A)** Boxplot analysis of two AD datasets (GSE28146 and GSE48350) before and after removal of batch effect. **(B)** Soft threshold powers analyzed by the unscaled fitting index (*β*) and average connectivity. **(C)** Dendrogram was generated using a hierarchical clustering method and dynamic tree algorithm. **(D)** Module-trait correlation heatmap showing associations between co-expression modules and AD clinical phenotypes. Red indicates positive correlation; blue denotes negative correlation.

Weighted gene co-expression network analysis was utilized to investigate gene modules associated with AD. The GEO datasets GSE28146 and GSE48350, generated on the same annotation platform, were combined into a matrix for subsequent WGCNA analysis following the elimination of batch effects. The combined dataset included 41 samples from AD patients and 50 from normal controls. Quality control assessment of the integrated dataset was visualized through boxplots (Figure 2A), confirming comparable expression distributions across batches. Next, the scale-free fit indices and average connectivity were evaluated under different soft threshold powers, with *β* = 5 being selected as the most appropriate parameter (Figure 2B). In the following dynamic tree algorithm, the threshold was set to a truncation height of 0.75, and the minimum number of genes per module was set to 100, which ultimately yielded 10 different co-expressed gene modules (Figure 2C). Module–trait relationships were evaluated by Pearson correlation analysis between module eigengenes and AD clinical phenotypes (Figure 2D). Statistical analysis revealed that the blue module, containing 2,385 genes, exhibited the strongest correlation with AD (Supplementary Table S2), establishing it as the most biologically relevant module for downstream investigation.

### 3.2 Identification and functional analysis of DEGs in AD dataset

**Figure 3:**
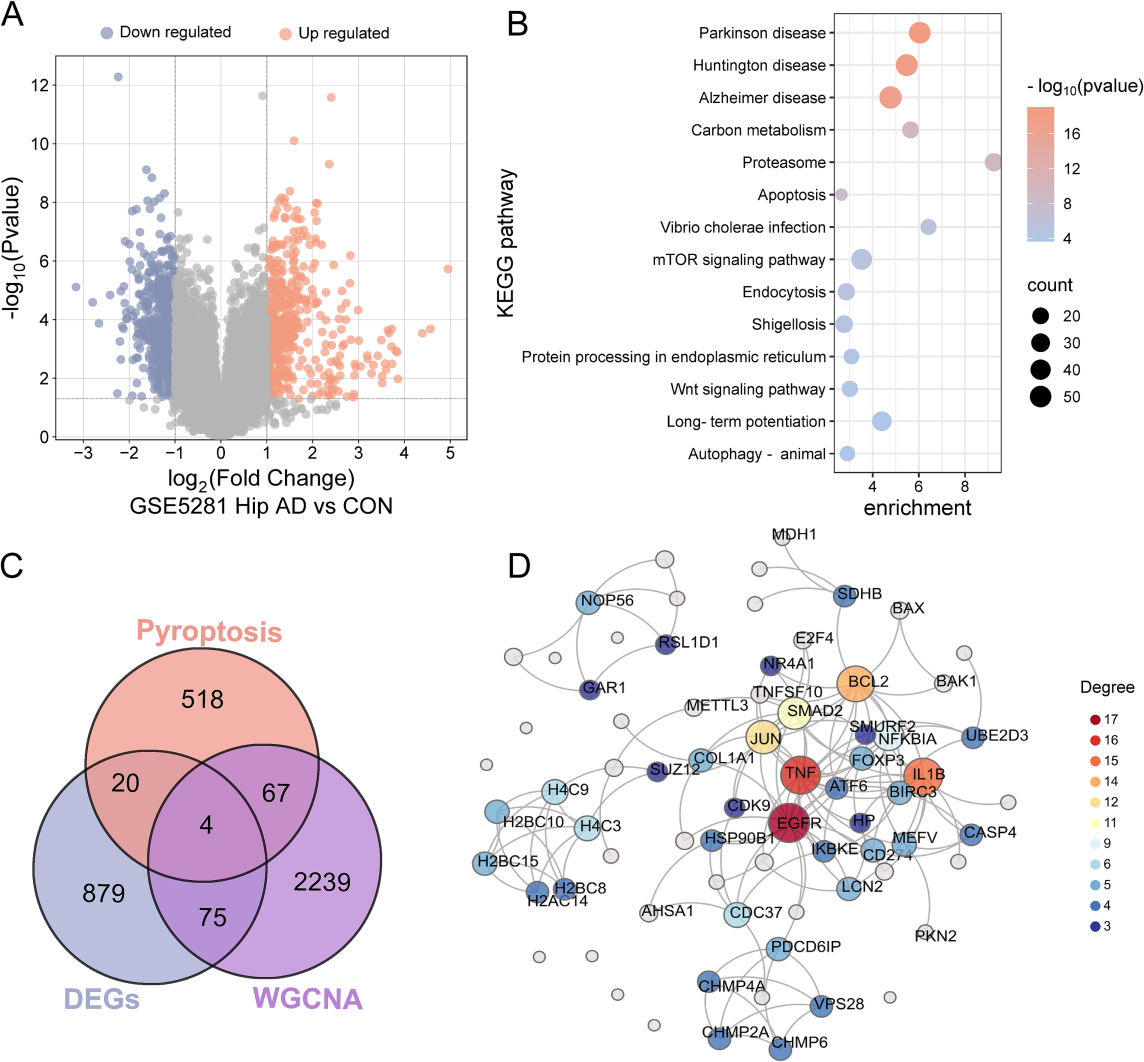
Identification and functional analysis of DEGs. **(A)** Volcano plot of DEGs in hippocampal tissues from GSE5281 (AD vs. control). **(B)** Bubble map of KEGG pathway enrichment analysis for DEGs, the enrichment scores, gene counts, and *p*-values were presented. **(C)** A Venn diagram of the gene list from pyroptosis, DEGs and WGCNA AD module genes, resulting in a total of 91 pyroptosis genes related to AD. **(D)** PPI network showed the interactions of the pyroptosis in AD. The names of the gene with connection degrees >2 or the combined scores > 0.9 were plotted, and the rest were not presented in the net.

Differential expression analysis was performed on hippocampal tissue samples from the GSE5281 dataset to identify AD-associated genes. After quality control verification via principal component analysis (PCA) and boxplots (Supplementary Figures S1A,B), 1,063 DEGs, of which 500 genes were upregulated and 563 genes were downregulated, were identified under thresholds of the fold change (FC) > 2 and *p*-values <0.05 (Figure 3A and Supplementary Table S3). Kyoto Encyclopedia of Genes and Genomes (KEGG) pathway results revealed that these DEGs were enriched in the pathways of neurodegeneration-multiple diseases, such as Parkinson’s or Alzheimer’s disease, and the pathways of cellular processes, such as apoptosis and autophagy (Figure 3B). Gene ontology (GO) analysis suggested that DEGs were enriched concerning neuron projection development, synapse, and transcription factor binding (Supplementary Figure S1C).

Given the established role of apoptosis or autophagy in AD pathogenesis (Zhang and Dai, 2024), we hypothesized that pyroptosis—a gasdermin-mediated programmed cell death—might contribute similarly. A total of 609 PRGs were obtained from the GeneCards database, and then an intersection analysis was conducted to ascertain the overlap between the 609 PRGs and 1,063 DEGs or the 2,385 blue module genes from WGCNA. Cross-analysis identified 91 PRGs in AD, and it was noteworthy that four genes (EGFR, NDUFA13, PKN2, and SUZ12) were presented in both the DEGs and the disease module of WGCNA (Figure 3C and Supplementary Figure S1D). The PPI network of the 91 pyroptosis-related AD genes was constructed using the String database and visualized by Chiplot (Figure 3D). Hub genes were prioritized with degree centrality >2 and combined interaction scores >0.9, yielding 47 key pyroptosis-related AD genes (Supplementary Table S4). The hierarchical clustering of these genes demonstrated distinct expression patterns (Supplementary Figure S1E).

### 3.3 Multiple machine learning algorithms identify hub genes of pyroptosis in AD

**Figure 4:**
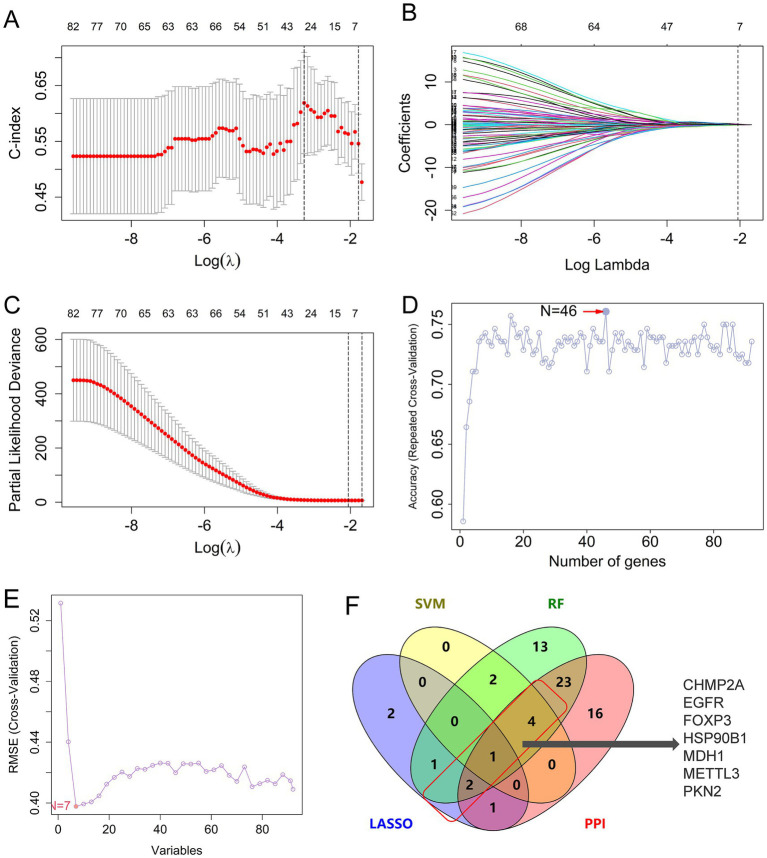
Identification of hub genes of pyroptosis in AD by machine learning. **(A–C)** The c-index model **(A)** and coefficients **(B)** and the partial likelihood deviances **(C)** of different genes varied with different log(lambda) in the LASSO algorithm. **(D)** Results of the RF algorithm: the accuracy was calculated as the number of genes changed. **(E)** The RMSE values in the SVM analysis. **(F)** Venn diagram intersecting LASSO, RF, SVM-derived genes, and PPI network candidates. LASSO, the least absolute shrinkage and selection operator; SVM, support vector machine; RF, random forest algorithm; PPI, protein–protein interaction.

To identify the hub genes with high diagnostic value among the 91 pyroptosis-related AD genes, three machine learning algorithms (LASSO, SVM, and RF) were applied using the integrated dataset from GSE28146, GSE48350, and GSE5281. In the LASSO analysis, the c-index model was employed to assess the predictive capacity of the model. At *λ* = 7, the c-index exhibited the greatest magnitude, the coefficient demonstrated convergence to 0, and the partial likelihood deviance exhibited a tendency toward 0, indicating that the model exhibited optimal predictive efficacy (Figures 4A–C). In the RF model, the highest level of accuracy was achieved when the number of genes increased to 46 (Figure 4D). In the SVM algorithm for prediction, the minimum deviation between the predicted and actual values was observed when the root mean square error (RMSE) reached its minimum and seven genes were used as features (Figure 4E). Consensus hub genes were defined as genes selected by ≥2 algorithms and intersected with PPI network-derived candidates, resulting in seven pyroptosis-AD hub genes: CHMP2A (the charged multivesicular body protein 2A), EGFR (epidermal growth factor receptor), FOXP3 (forkhead box P3), HSP90B1 (heat shock protein 90 beta family member 1), MDH1 (malate dehydrogenase 1), METTL3 (methyltransferase 3), and PKN2 (serine/threonine protein kinase C-related kinase 2) (Figure 4F and Supplementary Table S4). Among them, CHMP2A was the only gene that appeared in all three algorithms; EGFR, FOXP3, MDH1, and PKN2 were shortlisted in the RF and SVM models, while HSP90B1 and METTL3 were selected in both RF and LASSO algorithms.

### 3.4 Analysis of immune cell infiltration in AD

**Figure 5:**
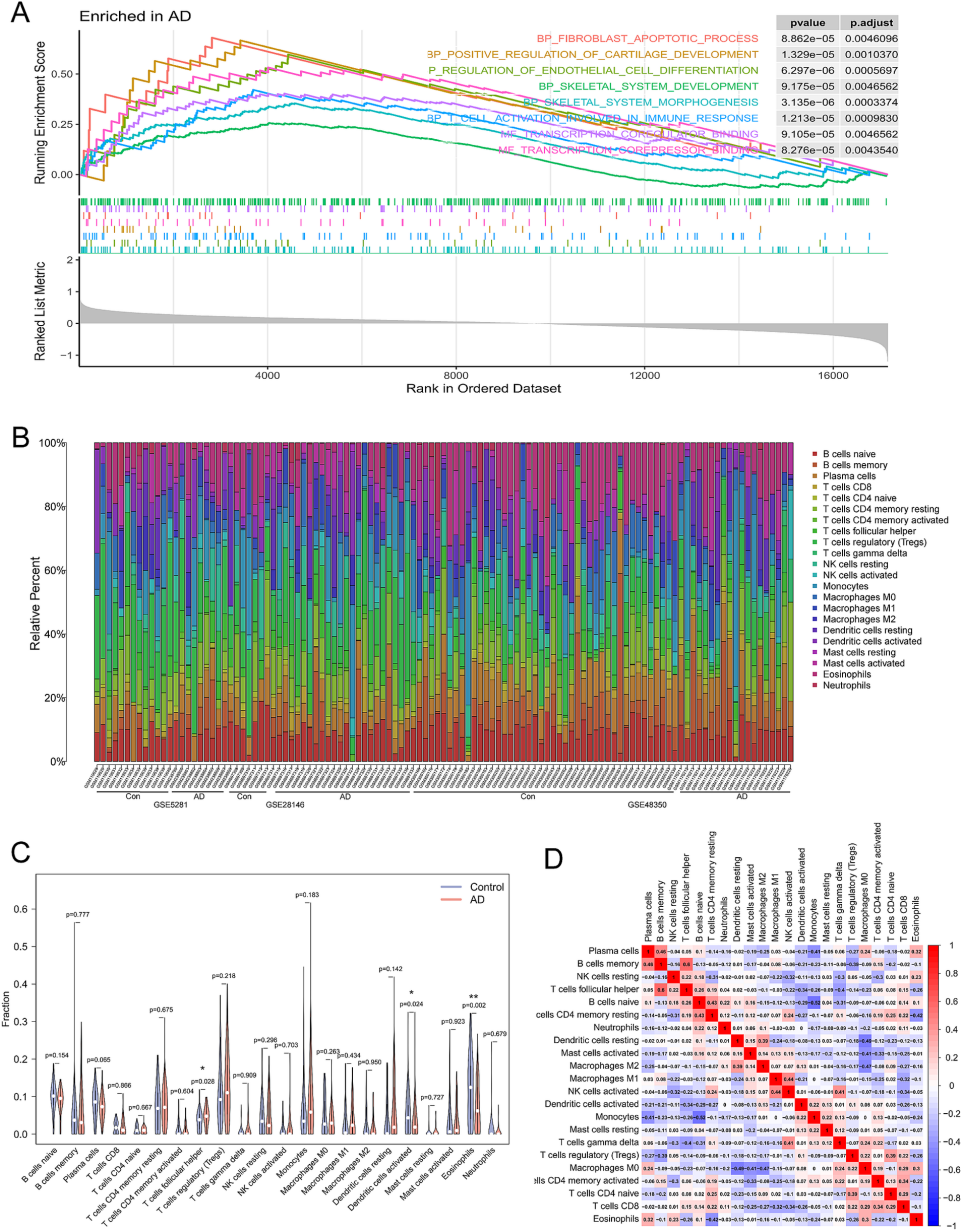
Assessment and visualization of immune cell infiltration. **(A)** GSEA was performed using the combined AD dataset (GSE28146, GSE48350, and GSE5281) with the GO terms as the gene set for enrichment. **(B)** The boxplot diagram illustrates the proportion of distinct immune cell types, whereas the heat map presents a summary of immune infiltration scores between AD patients and control. **(C)** The differences in immune infiltration of the 22 immune cells between AD (red) and normal (blue) controls from the combined dataset and *p-*values were shown as *p < 0.05, **p < 0.01. **(D)** The heat map shows the correlation infiltration of innate immune cells by CIBERSORT.

Gene set enrichment analysis revealed significant upregulation of the IMMUNE_RESPONE pathway in the AD group compared to the control (Figure 5A). Then, the CIBERSORT algorithm was employed to explore the immune microenvironment landscape of 22 immune infiltrating cells. Figure 5B illustrates the relative abundance of the immune infiltrating cells in AD and normal samples. The results of immune infiltration level revealed that eosinophils and dendritic cells were significantly downregulated in AD (*p* = 0.002, *p* = 0.024), whereas T cells follicular helper was upregulated in AD compared to the control (*p* = 0.028) (Figure 5C). Furthermore, to elucidate the interrelationships between these cells, a Pearson’s correlation analysis was conducted (Figure 5D), which provides insight into the complex network dynamics of the immune infiltrate in AD. These results confirmed the correlation between the pyroptosis-related genes and immune response.

### 3.5 Correlation analysis of the pyroptosis-AD hub genes and immune infiltration

**Figure 6:**
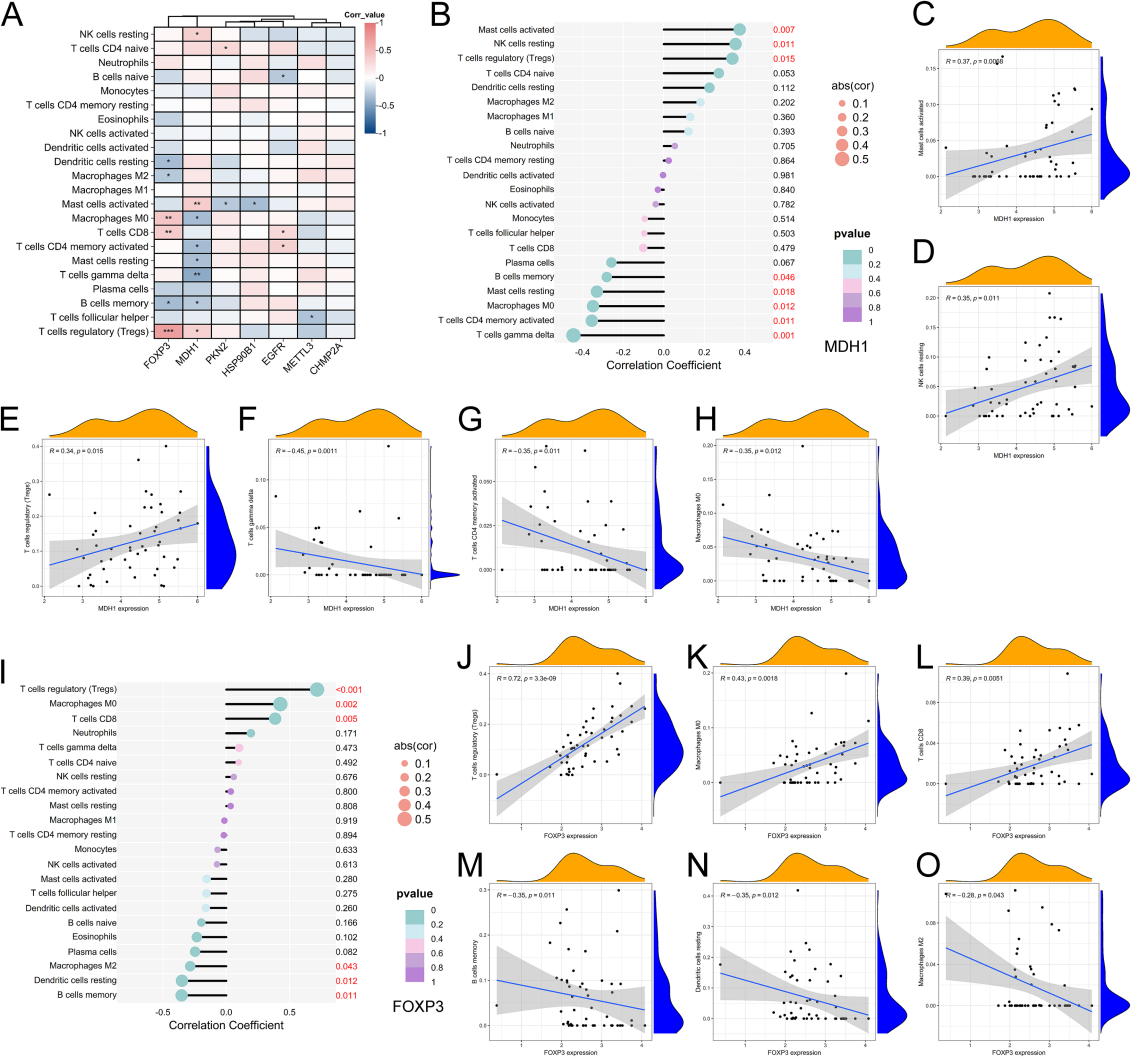
Correlation analysis between pyroptosis-AD hub genes and immune cell infiltration. **(A)** Heatmap showed the correlation and p-values of 22 immune infiltrating cells and pyroptosis-related genes. The red indicated a positive correlation, whereas the blue represented a negative correlation, and *p-*values were shown as *p < 0.05, **p < 0.01, ***p < 0.001. **(B)** Correlation analysis between MDH1 and infiltrating immune cells. **(C–H)** Correlation scatter plots between the expression of MDH1 and immune cells presented significance. **(I)** Correlation analysis between FOXP3 and infiltrating immune cells. **(J–O)** Correlation scatter plots between the expression of MDH1 and immune cells presented significance.

The relationship between the seven pyroptosis-AD hub genes and the 22 immune cells was then investigated. Based on the correlation and significance of these relationships, correlation heatmap was produced (Figure 6A). The results evidenced a correlation and statistical significance (*p* < 0.01) in the four types of immune cells [mast cells activated, regulatory T cells (Tregs), macrophages M0, and T cells CD8]. Among the genes, MDH1 and FOXP3 were found to be significantly correlated with multiple immune cell types (Figures 6B,I). Specifically, MDH1 was positively correlated with mast cells activated, regulatory T cells, and NK cells resting (Figures 6C–E) while exhibiting a negative correlation with T cells gamma delta, T cells CD4 memory activated, and macrophages M0 (Figures 6F–H). FOXP3 demonstrated a positive relationship with regulatory T cells (Tregs), macrophages M0, and T cells CD8 (Figures 6J–L). Conversely, it was negatively correlated with B cells memory, dendritic cells resting, and macrophages M2 (Figures 6M–O). These two genes both exhibited a positive correlation with regulatory T cells (Tregs). In addition, the remaining five genes were correlated with individual types of immune cells, as illustrated in their respective correlation plots (Supplementary Figure S2).

### 3.6 Validation of the pyroptosis-AD hub genes by the AD gene characterization and cross-platform expression

To ascertain the consistency of the seven pyroptosis-AD hub genes derived from machine learning algorithms with the characteristics of AD-related genes, the CFG method provided by the AlzData database was employed. This method quantifies gene-AD associations using a score from 0 to 5. A higher value indicates a greater relationship to AD. The results showed that the CFG values of EGFR and HSP90B1 up to 3, and CHMP2A, FOXP3, MDH1, and METTL3 attained 2 points (Supplementary Figure S3A). Subsequently, the cross-platform normalized expression levels of these genes in multiple brain regions were examined (Supplementary Figure S3B). The generated results demonstrated that five genes were significant in the AD sample; four of them (CHMP2A, EGFR, MDH1, and PKN2) exhibited differences (*p* < 0.05) in cross-platform expression level in AD tissues, while HSP90B1 showed alterations in independent dataset GSE5281. The remaining genes (FOXP3 and METTL3) did not show differences in any of the datasets in this database. Consequently, five genes (CHMP2A, EGFR, MDH1, PKN2, and HSP90B1) with robust evidence of AD-specific dysregulation were prioritized for subsequent regulatory network construction and *in vivo* validation in APP/PS1 mice.

### 3.7 Validation of the pyroptosis-AD hub genes in AD mice

**Figure 7:**
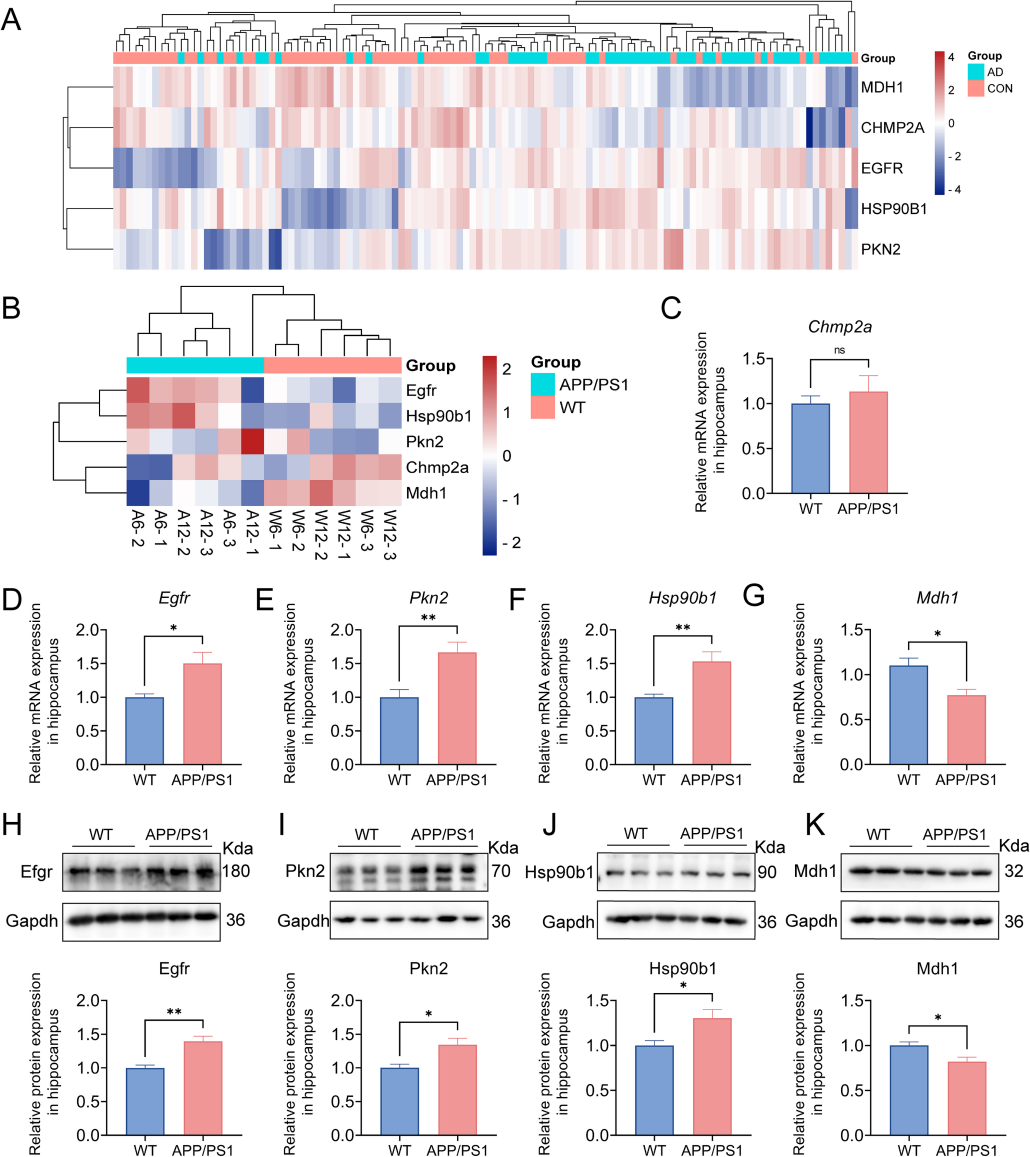
Validation of the pyroptosis-AD hub genes at the level of RNA and protein in AD mice. **(A)** A hierarchical clustering heatmap based on the normalized expression of the five pyroptosis-AD genes in the combined dataset. **(B)** A clustering heatmap was constructed based on the normalized expression of the five pyroptosis-AD genes in the 6- and 12-month-old APP/PS1 and control mice. The 6- and 12-month-old APP/PS1 or WT mice were abbreviated as A6 and A12 or W6 and W12, respectively. **(C–G)** qPCR validation of mRNA expression of the pyroptosis-AD hub genes (Chmp2a, Egfr, Pkn2, Hsp90b1, and Mdh1, respectively) between the 12 months APP/PS1 and wild-type (WT) mice. Data are mean ± SEM (*n* = 6 for WT, and *n* = 5 for APP/PS1 mice group, *p < 0.05, **p < 0.01, unpaired two-tailed *t*-test). **(H–K)** The cell lysates from the hippocampus of APP/PS1 and WT mice were prepared and blotted with anti-Egfr, Pkn2, Hsp90b1, and Mdh1, respectively (up). The relative protein expressions of Egfr, Pkn2, Hsp90b1, and Mdh1 were calculated using Gapdh as an internal reference (below). Data are mean ± SEM (*n* = 6 per group, *p < 0.05, **p < 0.01, unpaired two-tailed *t*-test).

In the preceding analysis, heatmaps and boxplots were generated to visualize the expression patterns of five dysregulated pyroptosis-AD hub genes in AD versus control samples (Figure 7A and Supplementary Figure S3C). To preliminarily validate the expression of these genes in AD mice, our dataset GSE242902 was explored, and the heatmap presented a comparison of the expression of these genes in APP/PS1 mice and WT mice at the ages of 6 and 12 months (Figure 7B). Then, qPCR analysis of hippocampal tissues further confirmed differential expression: Mdh1 was significantly downregulated (*p* = 0.0157), while Egfr (*p* = 0.0115), Pkn2 (*p* = 0.0067), and Hsp90b1 (*p* = 0.0038) were upregulated in the AD mice (Figures 7D–G). However, Chmp2a did not exhibit any significant differences in AD or control mice (Figure 7C). We proceeded to examine the alterations in protein levels of the four genes in the hippocampus of AD mice. The findings indicated that these protein changes were in accordance with those observed at the mRNA level, with Mdh1 exhibiting downregulation and Egfr, Pkn2, and Hsp90b1 demonstrating significant upregulation (*p* = 0.0012, 0.0194, 0.0118, and 0.0169, respectively) (Figures 7H–K). It was noteworthy that the trends of gene expression changes in AD mice were generally consistent with those observed in AD patients.

### 3.8 The diagnostic performance of pyroptosis-AD hub genes in the training and validation datasets

**Figure 8:**
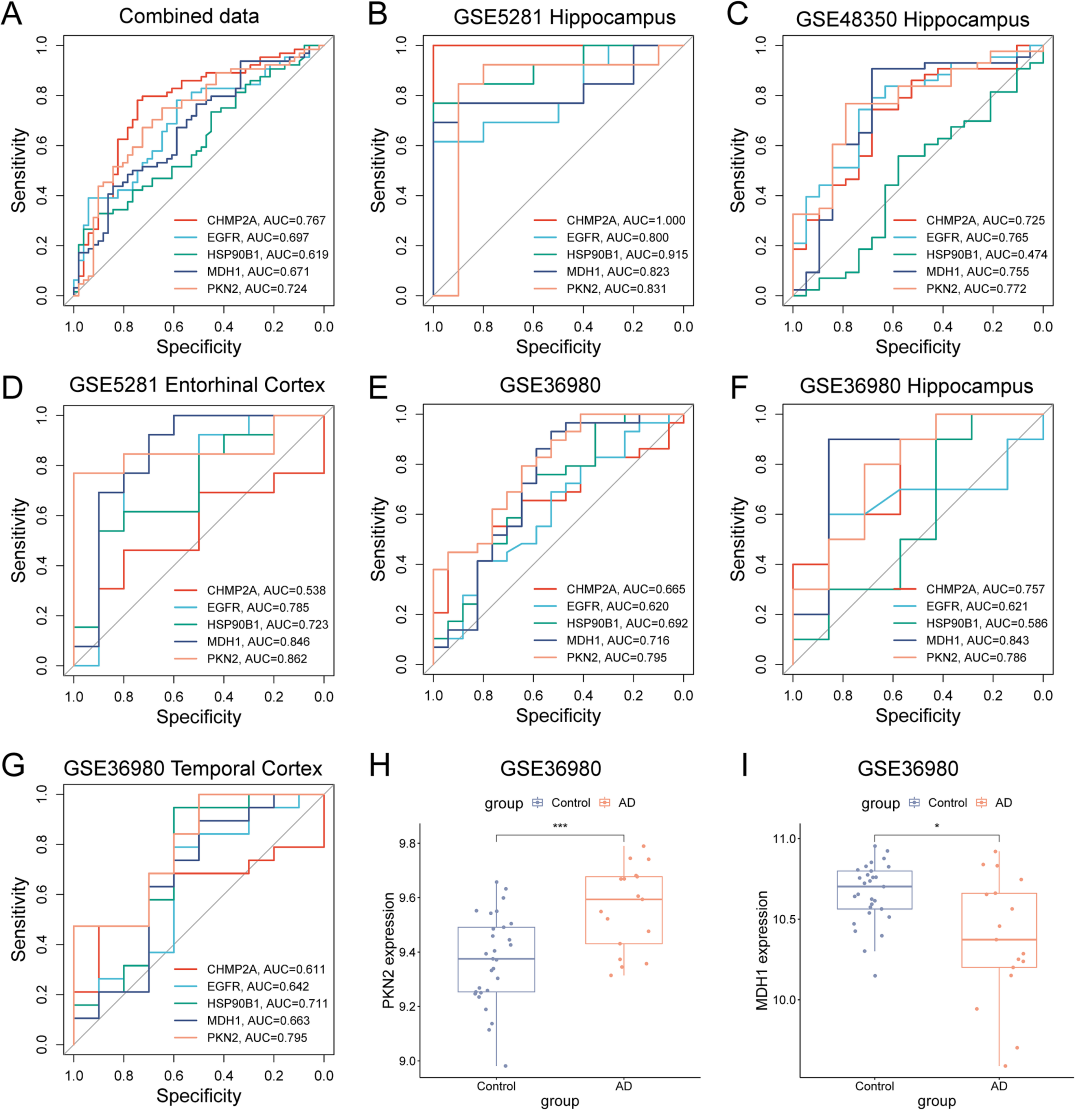
Screening and validation of candidate PRGs for the diagnosis of AD. **(A)** The ROC curve shows the diagnostic performance of the five feature genes in the combined dataset (training set). **(B,C)** ROC curves showing the diagnostic performance in the hippocampus of datasets GSE5281 **(B)** and GSE48350 **(C)**. **(D–G)** ROC curves show the diagnostic performance in the validation sets (entorhinal cortex of GSE5281, the hippocampus, or the temporal cortex data of GSE36980). **(H,I)** Differential expression of PKN2 and MDH1 in the GSE36980 (*p < 0.05, **p < 0.01, unpaired two-tailed *t*-test).

The diagnostic utility of these five pyroptosis-AD hub genes was systematically evaluated. In the combined training dataset, all five genes exhibited area under the receiver operating characteristic curve (AUROC) values exceeding 0.6, with PKN2 (AUROC = 0.724) and CHMP2A (AUROC = 0.767) demonstrating superior sensitivity and specificity (Figure 8A). The data employed for the combined analysis were selected from the hippocampal tissue expression profiles of those three datasets. Thus, we first selected the original individual datasets for ROC validation, and the result demonstrated that in the hippocampus tissue data from GSE5281, all five genes exhibited values exceeding 0.8, and in the data from GSE48350, four genes (in addition to HSP90B1) also remained above 0.7 (Figures 8B,C). To assess tissue-specific diagnostic robustness, we evaluated entorhinal cortex samples from GSE5281. MDH1 and PKN2 achieved exceptional AUROC values of 0.846 and 0.862, respectively, confirming their well specificity and sensitivity in both the cortex and the hippocampus of AD (Figure 8D). Additionally, we added the dataset GSE36980 as a validation set. The results demonstrated that all five genes manifested values exceeding 0.6, with MDH1 and PKN2 exhibiting values above 0.7 (Figure 8E). In the hippocampal tissue expression data of GSE36980, MDH1 and PKN2 performed even more effectively, with ROC values of 0.843 and 0.786, respectively (Figure 8F). Meanwhile, in the temporal cortex data of GSE36980, PKN2 continued to demonstrate superior performance (Figure 8G). A comparison of the expression of PKN2 or MDH1 in AD and control subjects in this dataset revealed a significant upregulation and downregulation of PKN2 and MDH1, respectively (Figures 8H,I), which was consistent with the results of previous gene expression validation studies in AD mice.

### 3.9 Construction of TFs and miRNAs regulatory networks of the pyroptosis-AD hub genes

To delineate the regulatory architecture governing pyroptosis-AD hub genes, we performed an integrated analysis of transcription factors (TFs), miRNAs. Through the target miRNA prediction process, 202 miRNAs for EGFR, 206 for PKN2, 12 for HSP90B1, 3 for MDH1, and 2 for CHMP2A were identified (Supplementary Table S5). Thirty-five of these miRNAs could co-regulate EGFR and PKN2, and four miRNAs were target genes for PKN2 and HSP90B1 (Supplementary Figure S4A). The same prediction process was performed on mouse genes and resulted in 15 miRNAs that could co-regulate Egfr and Pkn2 and one miRNA that was a common target gene of Pkn2 and Hsp90b1 (Supplementary Figure S4B). Notably, miR-335-3p emerged as a conserved regulator in both human- and mouse-derived genes, suggesting its pivotal role in AD-associated pyroptosis. For the identification of TFs, a total of 16 TFs were predicted for these hub genes (Supplementary Table S6), and the results suggested that MYC and SP1 were common TFs for the three upregulated genes (PKN2, EGFR, and HSP90B1), while FOXA1 for the two downregulated genes (MDH1 and CHMP2A) (Supplementary Figures S4C,D).

### 3.10 Construction of lncRNA regulatory network of the pyroptosis-AD hub genes

**Figure 9:**
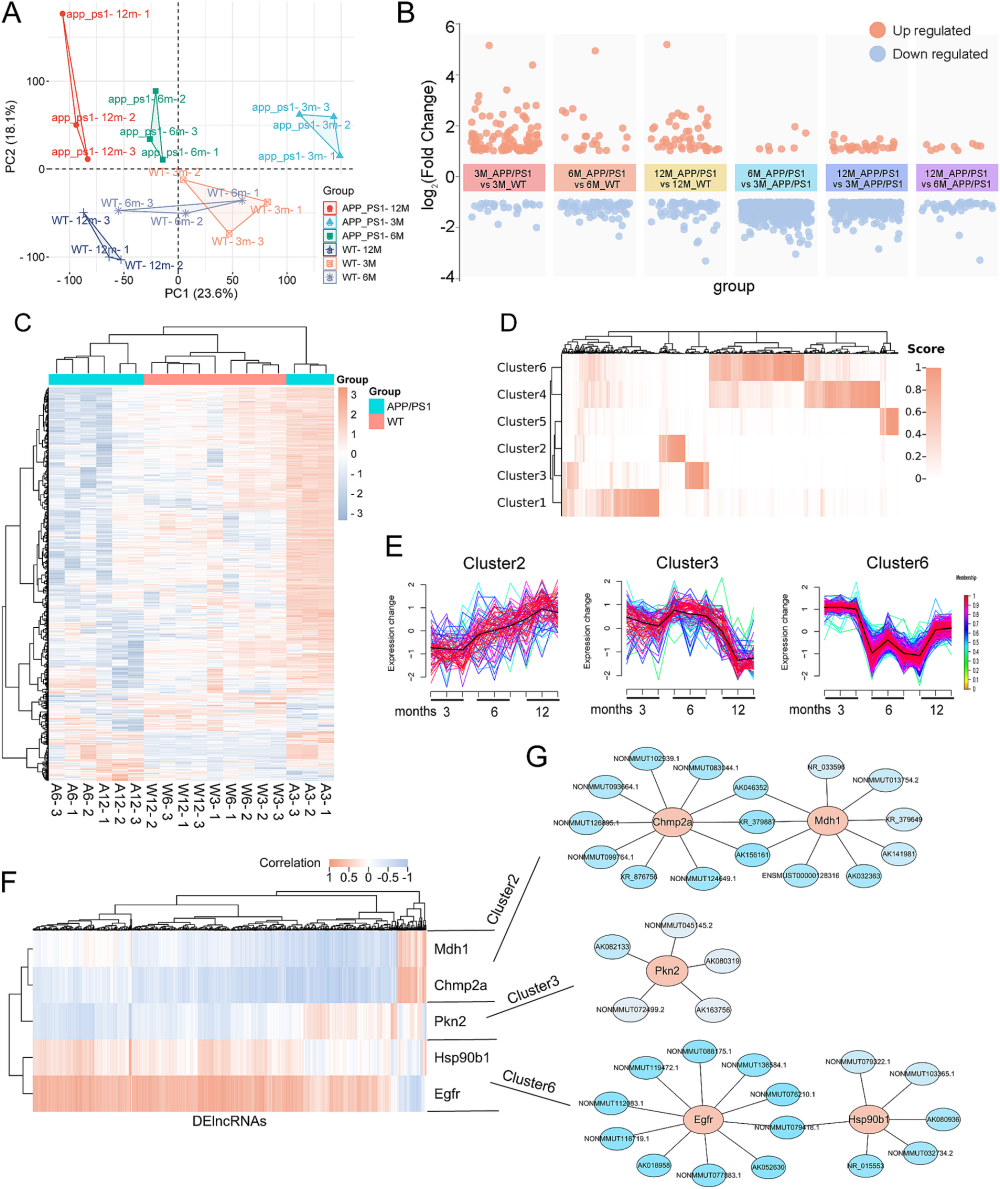
Construction of lncRNA regulatory network of the pyroptosis-AD hub genes. **(A)** PCA of lncRNAs expression profiles of the APP/PS1 and WT mice at the age of 3, 6, and 12 months. **(B)** Visualization of the clustered volcano diagram for the DElncRs from six different comparisons, including APP/PS1 mice vs. WT mice at the age of 3, 6, and 12 months and comparison of APP/PS1 mice between different ages. **(C)** A hierarchical clustering heatmap based on the normalized expression in all samples of DElncRs. The 3-, 6-, and 12-month-old APP/PS1 or WT mice were abbreviated as A3, A6, and A12 or W3, W6, and W12, respectively. **(D)** The clustered heatmap was produced based on the membership scores of the six clusters obtained by time series analysis. All the DElncRs and five pyroptosis-AD hub genes were clustered into six groups. **(E)** Line charts showed the relative expression trend in each cluster. The five pyroptosis-AD hub genes were divided into cluster 2 (Champ2 and Mdh1), cluster 3 (Pkn2), and cluster (Egfr and Hsp90b1). The horizontal axis represents a total of nine samples in the age 3-, 6-, and 12-month groups in turn. **(F)** The heatmaps of correlation analysis of the five pyroptosis-AD hub genes and DElncRs. **(G)** Regulatory networks constructed by the five pyroptosis-AD hub genes and their top10 (show all if the numbers of lncRNA less than 10) correlated lncRNAs (the ID of lncRNAs could be queried in the NONCODE, NCBI, or Ensemble databases).

To elucidate the temporal regulation of pyroptosis-AD hub genes during AD progression, we constructed lncRNA-mediated regulatory networks using transcriptomic data from APP/PS1 mice (GSE242902). This dataset profiled mRNAs and lncRNAs in hippocampal tissues at 3-, 6-, and 12-month time points. The expression of the five pyroptosis-AD hub genes has been presented (Figure 7B); we next analyzed the differential expression of lncRNAs in 3-, 6-, and 12-month-old APP/PS1 mice. The result of the principal component analysis of the samples was shown (Figure 9A). By setting up different subgroups for comparison, a total of 841 differentially expressed lncRNAs (DElncRs) were identified (duplicate DElncRs in multiple comparisons were integrated) (Figures 9B,C). Detailed group comparisons and the number of DElncRs were counted (only genes with a normalized expression value greater than 6 in any sample were retained) (Figure 9B and Supplementary Figure S5A).

Time series analysis was employed to investigate the potential regulatory relationship between these DElncRs and the five pyroptosis-AD hub genes with age. Genes exhibiting similar temporal variations were classified into the same cluster according to the membership scores (Figure 9D and Supplementary Table S7). To be precise, Chmp2a and Mdh1 were categorized in cluster 2, Pkn2 in cluster 3, and Egfr and Pkn2 in cluster 6 (Figure 9E and Supplementary Figure S5B). In parallel, a correlation analysis was employed to explore the correlation between these DElncRs and the pyroptosis-AD hub genes, which were plotted as a heatmap (Figure 9F). Then, the lncRNA-mRNA networks were constructed according to whether the target lncRNAs were in the same cluster and highly correlated with mRNA. The top 10 lncRNAs for each pyroptosis-AD hub genes were presented in the network (Figure 9G and Supplementary Table S8).

## 4 Discussion

Pyroptosis, a pro-inflammatory mode of programmed cell death, is characterized by the rupture of cellular membranes, leading to the release of inflammatory substances such as interleukin-1β (IL-1β) and IL-18 (Man et al., 2017). As a chronic neurodegenerative disease, AD is accompanied by persistent neuroinflammation driven by sustained microglial activation and cytokine release (De Strooper and Karran, 2016). The release of inflammatory factors, such as IL-1β, triggers a severe inflammatory cascade that ultimately results in neuronal damage or death (Allan et al., 2005). This indicates that neuroinflammation and pyroptosis may represent promising avenues for AD treatment. However, the roles and underlying mechanisms of pyroptosis in AD remain incomplete and require further investigation.

Recently, several studies have investigated the potential involvement of PANoptosis-related genes in AD (Zhang and Dai, 2024; Li et al., 2024; Lian et al., 2024). Similar to pyroptosis, PANoptosis is a type of programmed death that is characterized by features that include pyroptosis, autophagy, and necrotic apoptosis. Lian et al. (2024) identified necroptosis-specific molecular signatures associated with AD subtypes, demonstrating its regulatory role in necroptosis in AD progression. Moreover, the PANoptosis-related molecular subtypes identified by Li et al. (2024) were also associated with necroptosis and apoptosis, but not pyroptosis in AD. Nevertheless, differential expression of genes associated with pyroptosis is observed in AD (Xia et al., 2023). Therefore, a comprehensive investigation of the molecular signatures of pyroptosis-related genes and their regulatory networks in AD is essential for elucidating their diagnostic values and underlying mechanisms.

In this study, we leveraged hippocampal tissue expression data from multiple datasets to systematically analyze key pyroptosis-related genes in AD. Through the WGCNA and DEG analysis, we identified 71 and 24 PRGs associated with AD, respectively. Consequently, we proceeded to evaluate the hub genes among these 91 genes (four genes were shared in both WGCNA and DEGs results). By applying three machine learning algorithms and PPI functions analysis, seven candidate hub genes (CHMP2A, EGFR, MDH1, PKN2, HSP90B1, FOXP3, and METTL3) for pyroptosis were ultimately identified. Given that previous studies have confirmed immune cell dysregulation in AD (Yang et al., 2024; Liu et al., 2021), we used CIBEROST to analyze the immune infiltration patterns of PRGs in AD. Correlation analysis revealed that the seven pyroptosis-AD hub genes exhibited significant associations with three immune cell subtypes: eosinophils, dendritic cells, and T cells follicular helper. Notably, FOXP3 and MDH1 demonstrated superior performance, as they were associated with more than six types of immune cell infiltration. Both showed positive associations with regulatory T cells and memory B cells, indicating their potential contribution to cellular pyroptosis in AD. The infiltration increases of these two immune cells in AD patients have also been discussed (Lai et al., 2022).

We subsequently employed the method CFG analysis, proposed by AlzData, to ascertain the degree of association between our seven hub genes and AD. CFG analysis is an algorithm designed to prioritize AD candidate genes, and its ranking results are based on multiple AD disease data sources, which can be used to confirm the extent of the correlation between genes and AD (Xu et al., 2018). Indeed, all of the genes that we have identified through machine learning algorithms are associated with AD to varying degrees. In addition, the database provides cross-platform normalized expression profiles aggregated across multiple (up to 13) datasets, which means that it can be used to validate differential expression of genes (Lai et al., 2022; Wang et al., 2024). The results demonstrated that four (CHMP2A, EGFR, MDH1, and PKN2) of the seven genes exhibited differential expression in AD across multiple datasets. In addition to the hippocampus, differential expression was also observed in the entorhinal cortex and temporal cortex of AD patients compared to the controls (Supplementary Figure S3).

Indeed, these genes have been reported in AD studies, but their roles in pyroptosis in AD remain to be explored. CHMP2A belongs to the charged multivesicular body protein (CHMP) family, which is a component of ESCRT-III (endosomal sorting complex required for transport III) and is associated with neuronal development and autophagy (Vaz-Silva et al., 2018; Deneubourg et al., 2022). CHMP4B has recently been reported to be involved in the microglia pyroptosis in AD (Ding et al., 2024). MDH1 was identified as an indicator of microglia activation in AD, but specific mechanisms in AD are lacking (Pesamaa et al., 2023). PKN2 was thought to be involved in the neuroprotective role in hypoxia (Thauerer et al., 2014). EGFR has been relatively well-studied in AD, with a review suggesting that EGFR inhibition is critical for modulating the amyloid pathway as it contributes to microtubule stabilization and attenuates the secretion of pro-inflammatory molecules to reduce severe neuronal degeneration (Jayaswamy et al., 2023). For the other two genes, FOXP3 and METTL3, although no significant alterations were found in the cross-platform normalized expression profiles, their roles in AD have been reported. FOXP3’s participation in regulatory T cell process can regulate the microglial inflammatory response of AD (Yang et al., 2022), while METTL3 affects the development of AD through m6A methylation (Yang et al., 2023; Yin et al., 2023). Subsequently, we utilized 12-month-old APP/PS1 mice, which exhibit more pronounced AD-like neuropathological features than 6-month-old animals, to investigate the expression profiles of these pyroptosis-associated genes. Our findings demonstrated that the transcriptional alterations observed in these AD mice closely paralleled the molecular signatures observed in human AD patient datasets. However, unexpectedly, the changes in CHMP2A in AD mice were not significant, suggesting that a larger sample size may be required for more definitive conclusions. Furthermore, these genes demonstrated robust diagnostic utility, with ROC values exceeding 0.6 in the combined dataset. In particular, MDH1 and PKN2 exhibited exceptional performance across independent datasets and brain regions (hippocampus and cortex), underscoring their translational potential as AD biomarkers.

To elucidate the potential regulatory relationship between these PRGs in AD, we sought to elucidate the underlying regulatory network. Among the predicted microRNA regulators, miR-335-3p emerged as a critical candidate due to its conserved regulatory roles in human and murine EGFR and PKN2 expressions. Studies have reported that miR-335-3p is involved in cell differentiation and apoptosis (Kay et al., 2019; Sun et al., 2022), suggesting its potential function in pyroptosis modulation. LncRNA are RNAs with regulatory functions widely expressed in the brain (Wu et al., 2025; Liu et al., 2022). To explore the dynamic connection between pyroptosis genes and DElncRs, we employed the Mfuzz algorithm to identify genes with similar expression patterns. Using time series analysis and correlation analysis, we constructed the lncRNA-mRNA regulatory network in AD of these genes. LncRNAs in the hub network can be taken to play pivotal roles in regulating pyroptosis.

While this study provides novel insights into pyroptosis regulation in AD, several limitations warrant acknowledgment. Our study is just a pioneering study, and further validation experiments are necessary to elucidate how these pyroptosis-related genes play a role in AD. Although we validated the expression using external datasets and mouse tissues, additional testing in human brain tissue is essential to substantiate these findings. Furthermore, we have conducted preliminary investigations into the potential regulatory pathways of the PRGs, and the mechanisms of these genes acting in AD require further exploration.

## 5 Conclusion

In summary, our study represents a pioneering effort in identifying hub genes associated with pyroptosis in AD. By employing multiple transcriptomics and machine learning, we have successfully identified seven key genes. Of these, MDH1 and PKN2 demonstrated superior performance in the subsequent comprehensive evaluation, which was achieved through immune cell infiltration, ROC curves, and experimental expression validation. Furthermore, we have established a regulatory network of these genes. These findings not only provide novel insights into the pivotal genes associated with pyroptosis but also offer potential molecular targets for the diagnosis and treatment of AD.

## Data Availability

Publicly available datasets were analyzed in this study. This data can be at: https://www.ncbi.nlm.nih.gov/geo/ and their accession numbers are GSE28146, GSE48350, GSE5281, GSE36980, and GSE242902.

## Footnotes

^1^ https://cn.string-db.org/

^2^ https://www.chiplot.online/
