# Supplementary material for: Identification and validation of pyroptosis-related genes in Alzheimer’s disease based on multi-transcriptome and machine learning
Source: Front Aging Neurosci. 2025 May 14;17:1568337. doi: 10.3389/fnagi.2025.1568337 (PMC12116433; doi:10.3389/fnagi.2025.1568337)
Supplement: Supplementary file 2 [file Table_2.docx]

**Identification and validation of pyroptosis-related genes in Alzheimer’s disease based on multi-transcriptome and machine learning**

**Supplementary Figures**

**Supplementary Figure** **S1**


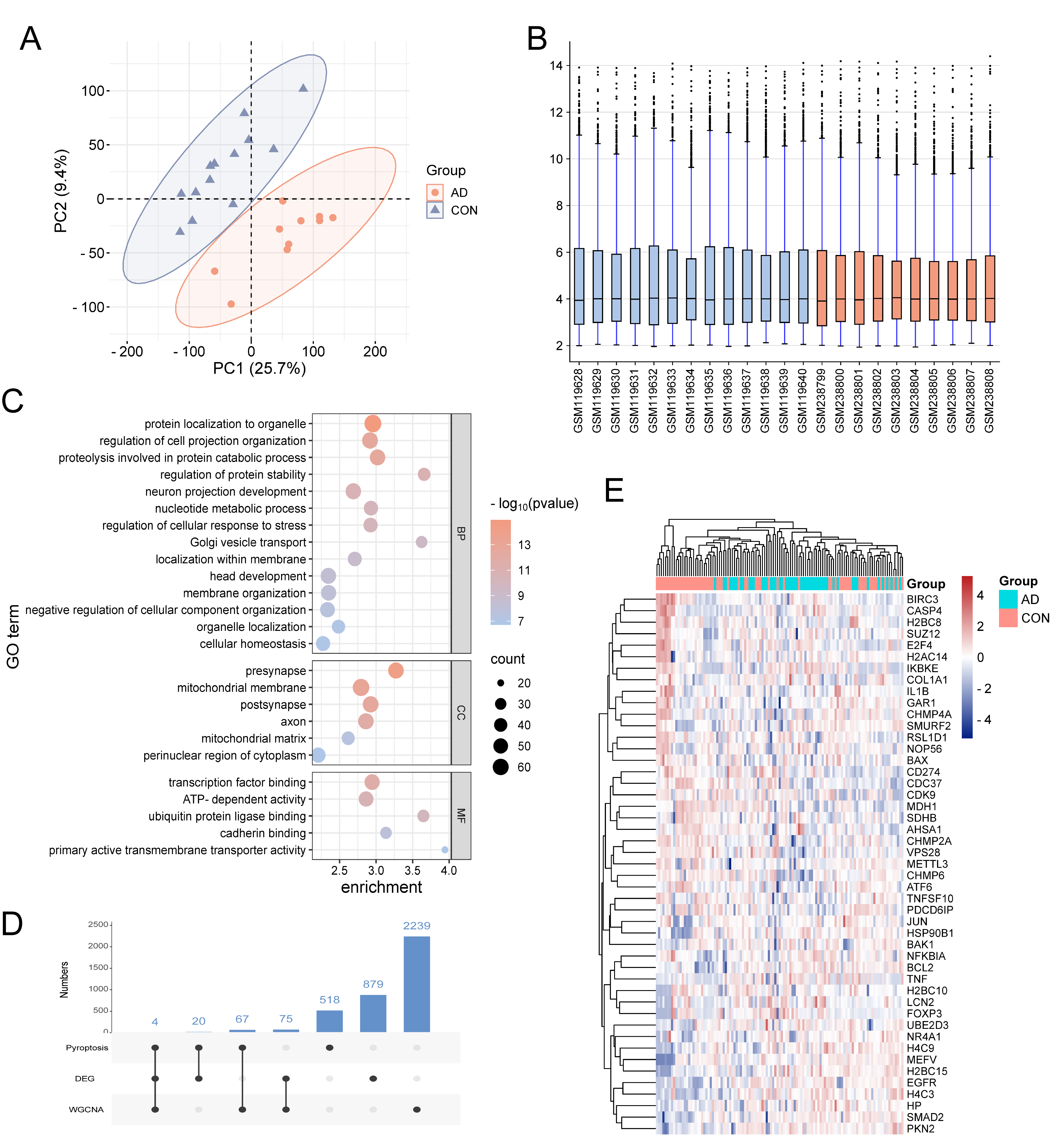


**Fig. S1 Identification and functional analysis of DEGs. (A-B)** The results of principal component analysis (PCA) (A) and boxplot (B) of the expression from the AD and control hippocampus of GSE5281. **(C)** Bubble map of GO analysis (BP, Biological Process; MF, Molecular Function; CC, Cellular Component) for DEGs, the enrichment scores, gene counts and *p*-values were presented. **(D)** The intersect of the gene list from pyroptosis, DEGs and WGCNA AD genes. **(E)** A hierarchical clustering heatmap based on the normalized expression of combined dataset from the hippocampus tissue of GSE28146, GSE48350 and GSE5281.

**Supplementary Figure** **S2**


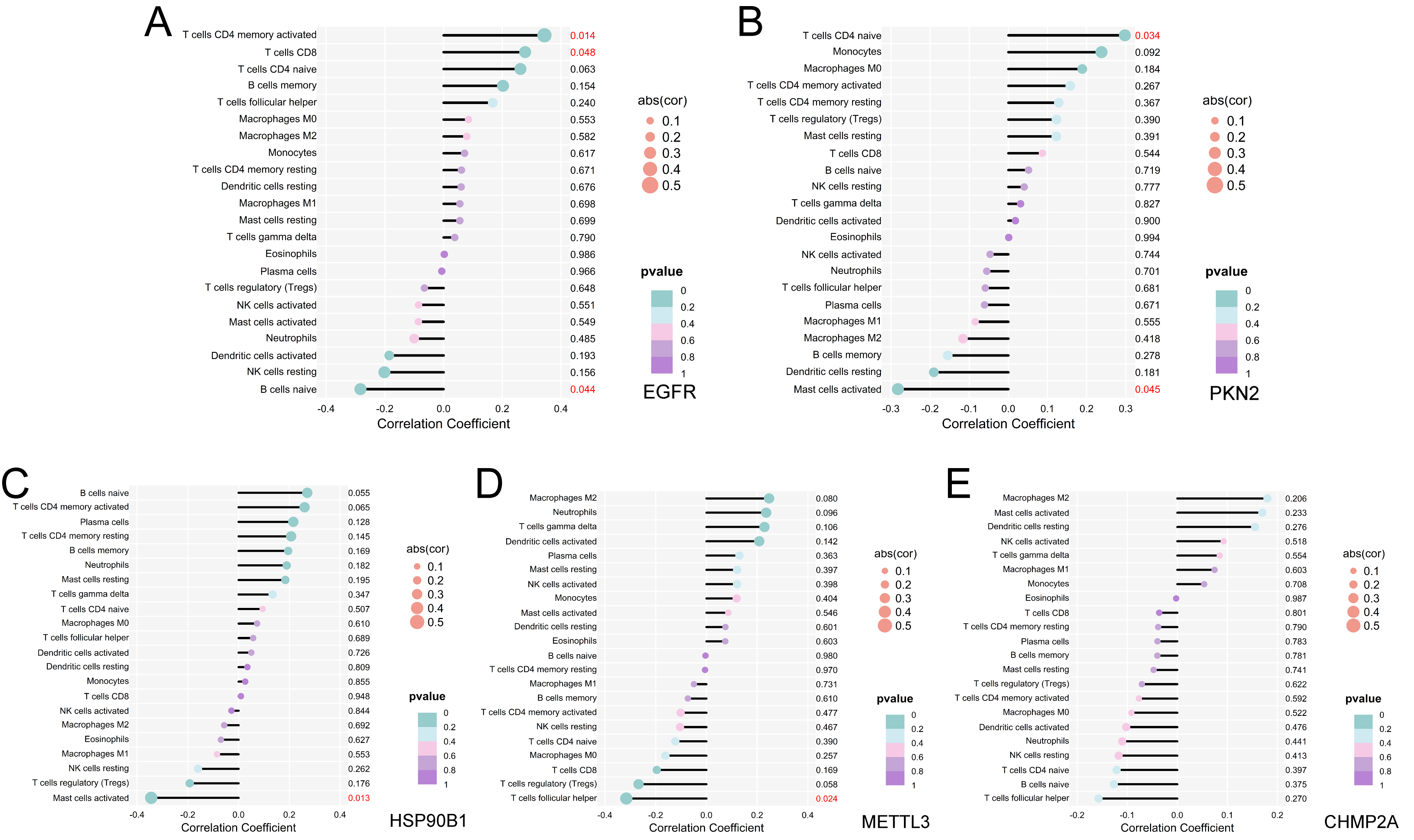


**Fig. S2 Correlation analysis between the pyroptosis-AD hub genes and immune cell infiltration. (A-E)** Correlation analysis between EGFR, PKN2, HSP90B1, METTL3, CHMP2A and infiltrating immune cells, respectively.

**Supplementary Figure** **S3**


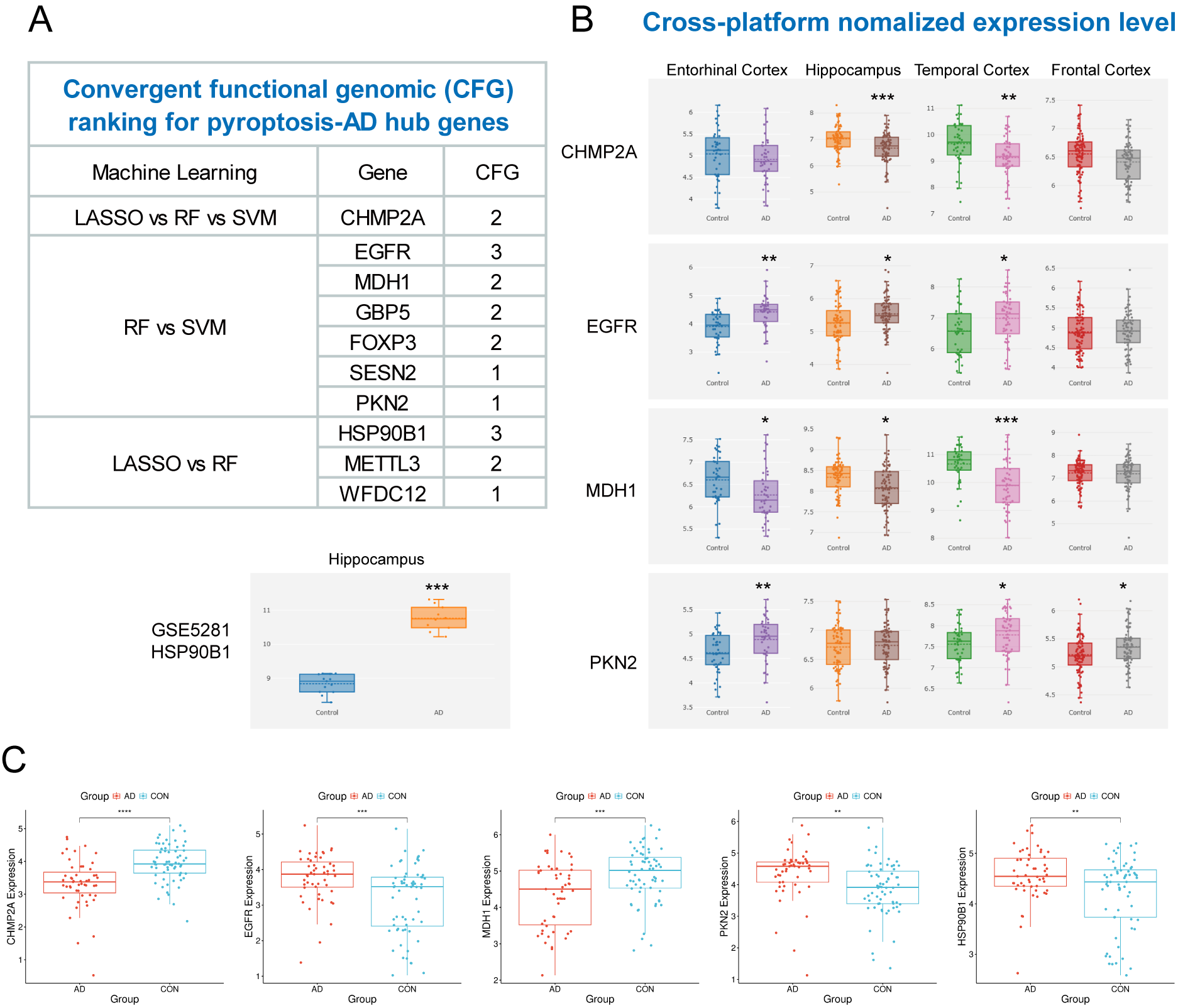


**Fig. S3 Identification of pyroptosis-AD hub genes by CFG analysis and cross-platform expression**. **(A)** The Convergent functional genomic (CFG) analysis result of the pyroptosis-AD hub genes identified by the machine learning methods. The CFG values range from 0 to 5, and a higher value means more relevant to AD. **(B)** The expression of the pyroptosis-AD hub genes from the cross-platform normalized expression in the AlzData database, and *p* values were shown as **P* <0.05, ***P* <0.01, ****P* <0.001. **(C)** The boxplot showed the expression of the pyroptosis-AD hub genes from the combined dataset, and *P-*values were shown as ***P* <0.01, ****P* <0.001.

**Supplementary Figure** **S4**


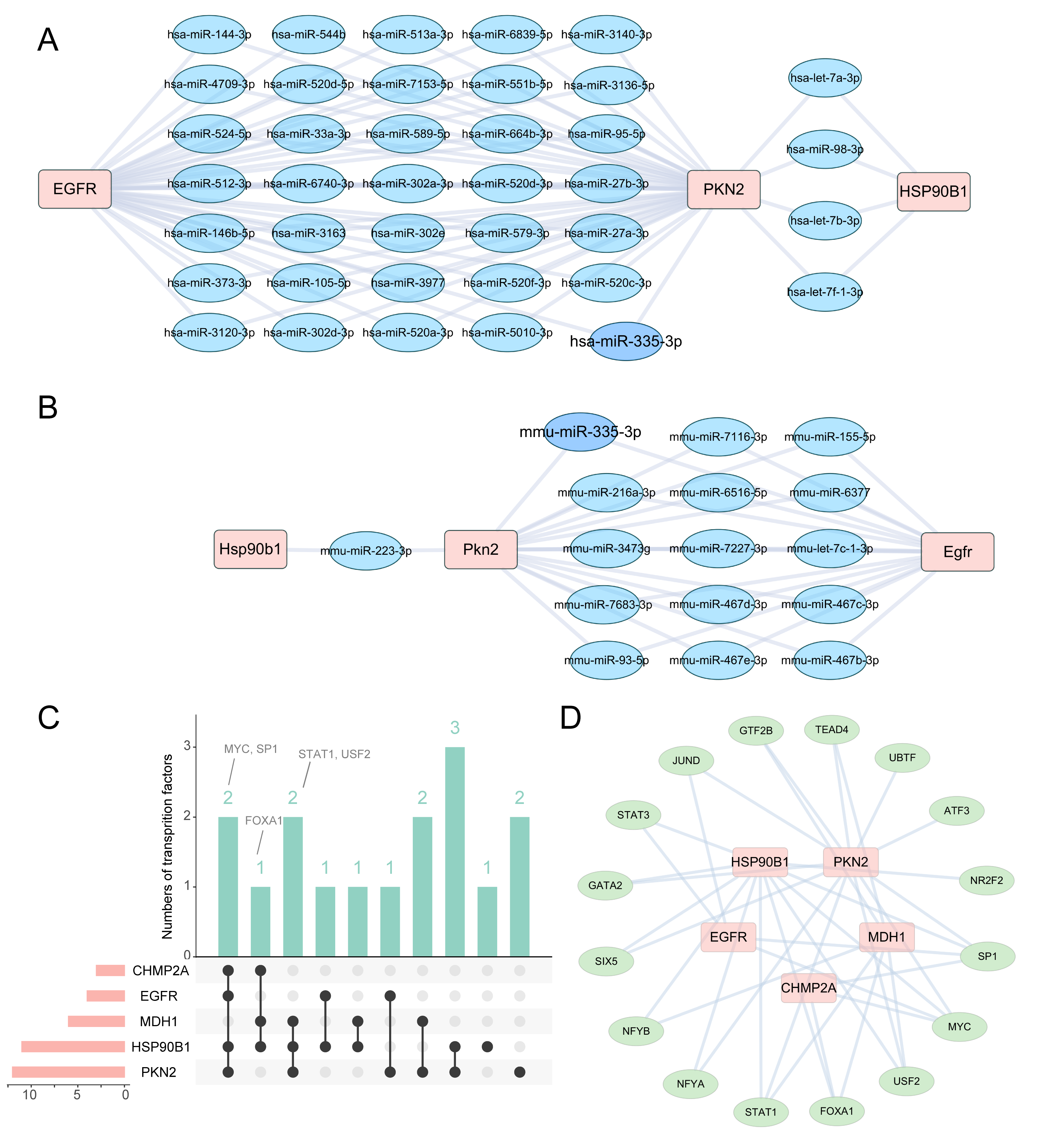


**Fig. S4 Construction of transcription factors and miRNA regulatory networks for the pyroptosis-AD hub genes**

**(A)** The network was constructed based on the identified the 35 miRNA targets of EGFR and 39 miRNA targets of PKN2 and 4 targets for HSP90B1. **(B)** The network were constructed by the 15 miRNA potiental targets of Egfr and 16 miRNA targets of Pkn2 and 1 targets for Hsp90b1. **(C-D)** Transcription factor interactions for these pyroptosis-AD hub genes demonstrated using upsetR (C) and network (D).

**Supplementary Figure** **S5**


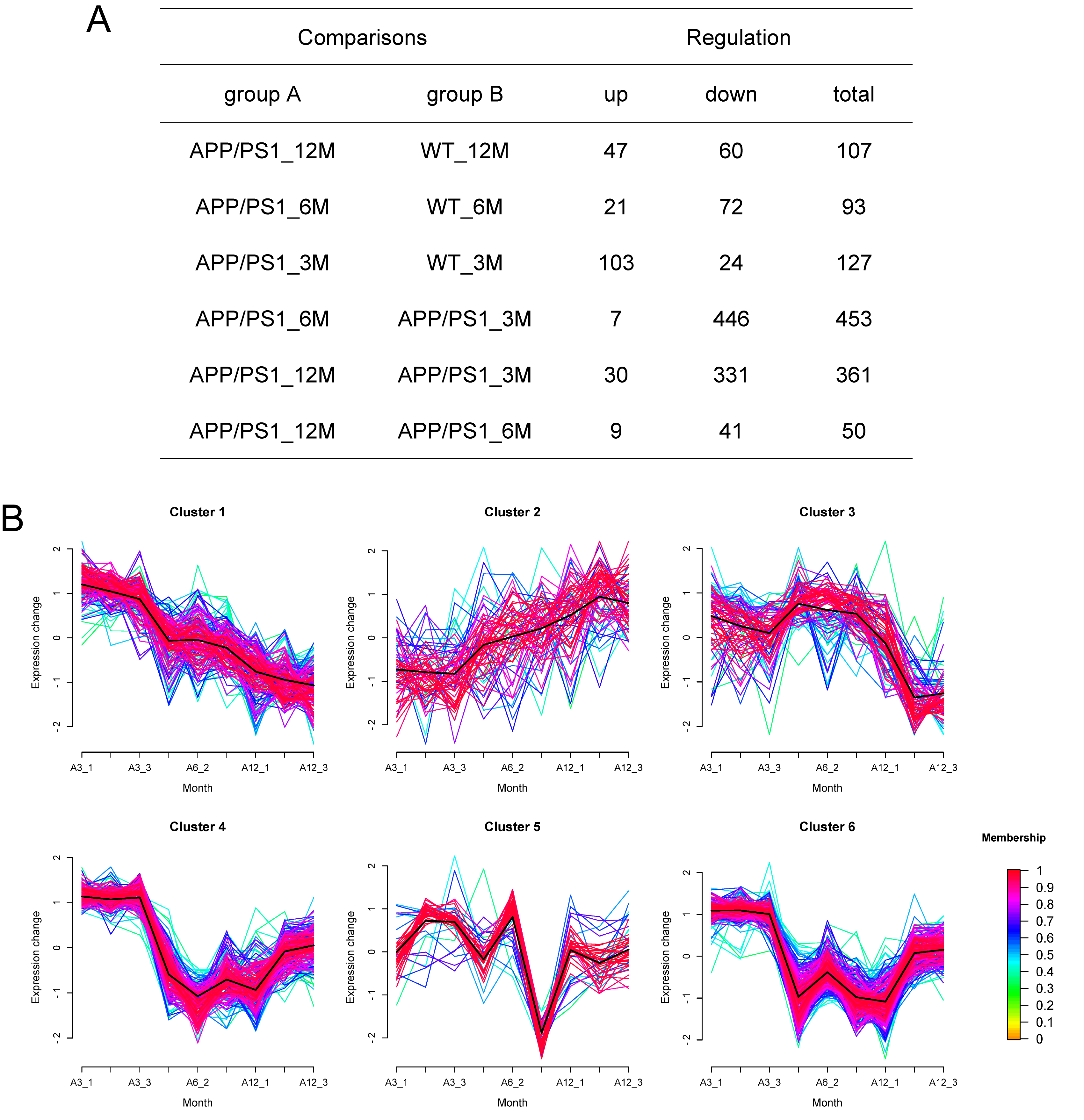


**Fig. S5 Differentially expressed lncRNA profiles and their expression trends. (A)** A summary table of differentially expressed lncRNAs in all the comparsions. **(B)** All the DElncRs and five pyroptosis-AD hub genes were clustered into 6 groups. Line charts showed the relative expression changes of genes in each cluster. The horizontal axis represents a total of nine samples at the age of 3-, 6-, and 12-month group in turn (*n* = 3 per group).
